# *Ex vivo* analysis of DNA repair targeting in extreme rare cutaneous apocrine sweat gland carcinoma

**DOI:** 10.18632/oncotarget.27961

**Published:** 2021-05-25

**Authors:** Rami Mäkelä, Ville Härmä, Nibal Badra Fajardo, Greg Wells, Zoi Lygerou, Olle Sangfelt, Juha Kononen, Juha K. Rantala

**Affiliations:** ^1^Misvik Biology Oy, Turku, Finland; ^2^University of Sheffield, Department of Oncology and Metabolism, Sheffield, UK; ^3^University of Patras, Laboratory of General Biology, Patras, Greece; ^4^Karolinska Institutet, Department of Cell and Molecular Biology, Stockholm, Sweden; ^5^Docrates Cancer Hospital, Helsinki, Finland

**Keywords:** cutaneous apocrine sweat gland carcinoma, *ex vivo* drug screening, DNA repair, *PALB2*, rare cancer

## Abstract

Cutaneous apocrine carcinoma is an extreme rare malignancy derived from a sweat gland. Histologically sweat gland cancers resemble metastatic mammary apocrine carcinomas, but the genetic landscape remains poorly understood. Here, we report a rare metastatic case with a *PALB2* aberration identified previously as a familial susceptibility gene for breast cancer in the Finnish population. As PALB2 exhibits functions in the BRCA1/2-RAD51-dependent homologous DNA recombination repair pathway, we sought to use *ex vivo* functional screening to explore sensitivity of the tumor cells to therapeutic targeting of DNA repair. Drug screening suggested sensitivity of the PALB2 deficient cells to BET-bromodomain inhibition, and modest sensitivity to DNA-PKi, ATRi, WEE1i and PARPi. A phenotypic RNAi screen of 300 DNA repair genes was undertaken to assess DNA repair targeting in more detail. Core members of the HR and MMEJ pathways were identified to be essential for viability of the cells. RNAi inhibition of RAD52-dependent HR on the other hand potentiated the efficacy of a novel BETi ODM-207. Together these results describe the first ever CAC case with a BRCAness genetic background, evaluate combinatorial DNA repair targeting, and provide a data resource for further analyses of DNA repair targeting in PALB2 deficient cancers.

## INTRODUCTION

Metastatic cutaneous apocrine gland carcinoma (CAC) is an extreme rare malignancy arising from a sweat gland with < 30 reported cases in the literature [1–3]. Majority of these reported cases are derived from the axilla with only a few cases originating from other regions of the integumentary system [4]. The tumorigenesis of these rare cancers is largely unclear, but histologically cutaneous apocrine gland carcinomas mimic metastatic apocrine breast cancer or apocrine carcinomas arising in ectopic breast tissue [5, 6]. To establish correct clinical diagnosis CAC thus needs to be distinguished from these other apocrine malignancies through detailed histological examination [7]. Although the reported local recurrence rate and lymph node metastasis for CAC cases is around 50%, reported mortality from the disease is low [2]. The treatment of choice for CAC is wide local excision with clear margins, with or without lymph node dissection [10]. There are currently no guidelines for treatment of widespread metastatic CAC [8–10]. However, for patients with metastatic disease radiotherapy and chemotherapy have been used as adjunctive treatments, but have shown little benefit on mortality [3, 9, 10]. Given the rarity of metastatic CAC tumors and the heterogeneity of the treatments used, the survival benefits of cytotoxic agents in treatment of metastatic CAC remains unclear, as does the use of targeted therapies, which have been reported for a few individual patients [11–14]. Therefore, there is a need for better understanding of the complex biology of cutaneous apocrine sweat gland carcinomas, including the molecular and genetic background of these malignancies to develop rationales for therapeutic strategies.

Here, we report a rare cutaneous apocrine sweat gland carcinoma case with widespread metastatic dissemination. We used multiomics methods for *ex vivo* analysis of the patient derived tumor cells with the aim to inform the treatment of the patient after relapse following 9 previous treatment regimens. Targeted exome sequencing identified a biallelic *PALB2* mutation, a *CHEK2* mutation and amplification of *MYC*. An *ex vivo* drug screen was performed to test sensitivity of the patient derived tumor cells to 165 anti-cancer drugs [15–17]. Altogether 48 drugs including multiple DNA repair targeting agents displayed higher efficacy than the 9 chemotherapies previously used to treat the patient. Of these, two different BET-bromodomain inhibitors; JQ1 and ODM-207 were the most potent drugs with a known DNA repair targeting mechanisms. To assess DNA repair pathways essential for the tumor cells and contributing to sensitivity/resistance of the tumor cells to BETi, we use *ex vivo* functional RNAi screening [18] to discover biological insights on the different DNA repair pathways with the PALB2 deficient cells. To extend the analysis beyond the patient derived cells, we go on to assess with publicly available drug screening data the correlation between efficacy of PARP inhibitors Olaparib, Talazoparib and BETi on 800 model cell lines divided to unaltered or PALB2 altered cell lines. Finally, we compare the frequency of mutations of the core HR genes *BRCA1/2*, *CHEK2* and *PALB2* in non-melanoma skin cancers. In summary, we identify that the BRCAness phenotype could be a pathogenic and targetable feature in subset of non-melanoma skin cancers and that PALB2 altered cells display increased sensitivity to BETi which may be further potentiated with combination of PARPi, supporting similar findings reported in other human cancers.

## RESULTS

### Targeted genomic profiling and *ex vivo* drug efficacy screening

**Figure 1:**
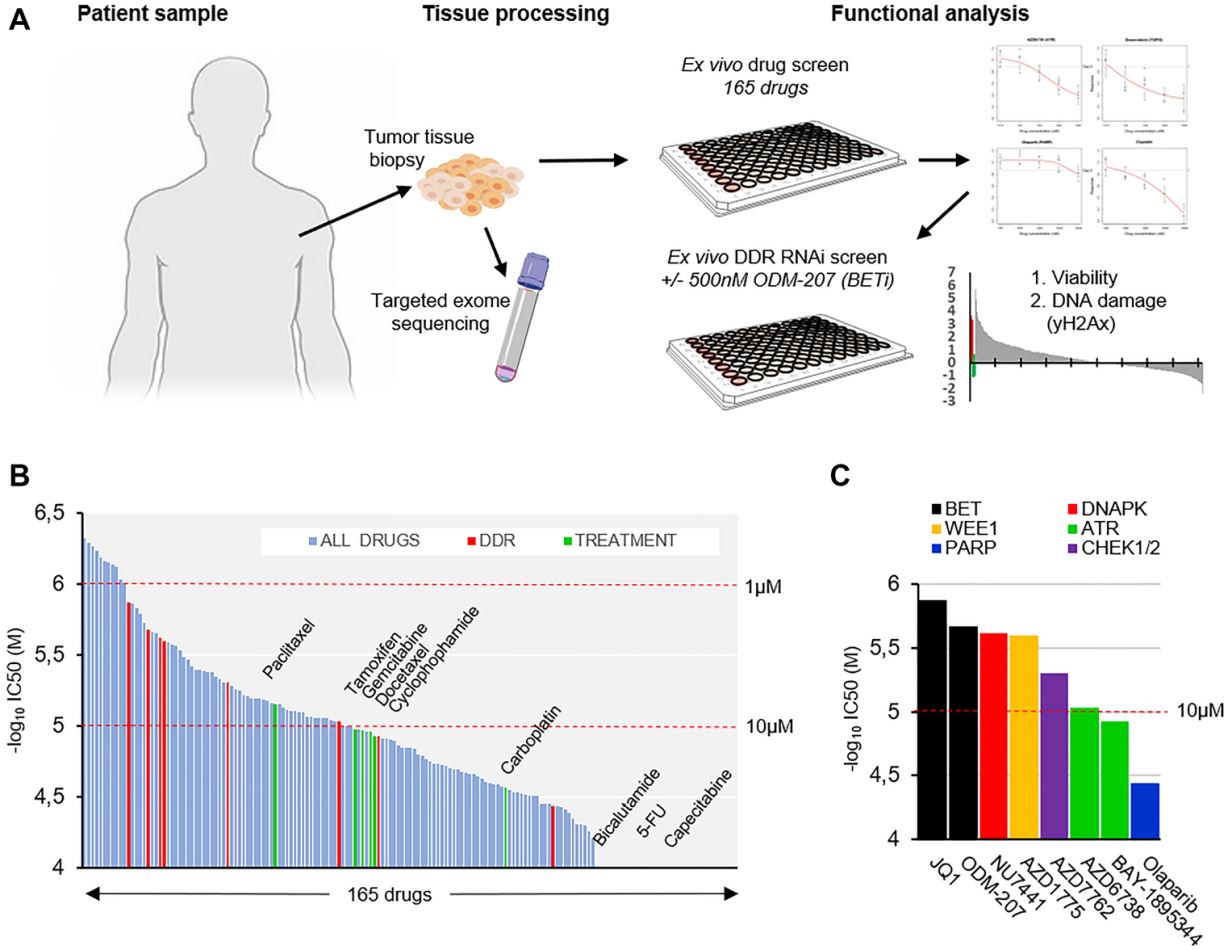
*Ex vivo* analysis of therapeutic strategies in a metastatic apocrine sweat gland cancer. (**A**) Schematic representation of the study strategy. Live tissue samples were used for targeted NGS profiling, *ex vivo* drug- and RNAi screening. (**B**) Waterfall plot of IC_50_ estimates of 165 drugs included in the drug screening. Drugs ordered according to –log_10_ of IC_50_ (molar) left to right. Drugs used previously to treat the patient shown in green and DDR targeting drugs in red. (**C**) Bar graph showing the IC_50_ of the included DDR targeting drugs in order of decreasing efficacy (–log_10_ of IC_50_(M)).

Prior to this study the patient’s disease had continued to progress through 9 different chemotherapy regimens. At this stage coarse needle tumor biopsies were obtained for the purpose of genetic profiling and *ex vivo* therapy efficacy screening (Figure 1A). Targeted DNA exome sequencing using the FoundationOne CDx™ test (Foundation Medicine, Inc) identified altogether 7 genomic aberrations. Among the 3 detected gene mutations two were truncating mutations in the *PALB2* and one a missense mutation in *CHEK2* gene. In addition to the three mutations, *MYC*, *LYN* and *RAD21* all located on Chr8q and *RPTOR* located on Chr17q25.3 were found to be amplified (Table 1).

**Table 1:** Genomic aberrations discovered in the patient tumor tissue

Gene	Effect	Impact	Copy #	Protein change
PALB2	FRAME_SHIFT	HIGH	Diploid	L531fs*30
PALB2	FRAME_SHIFT	HIGH	Diploid	F557fs*18
CHEK2	MISSENSE	HIGH	Diploid	I157T
MYC	AMPLIFICATION	HIGH	Gain	-
LYN	EQUIVOCAL AMPLIFICATION	MODERATE	Gain	-
RAD21	AMPLIFICATION	MODERATE	Gain	-
RPTOR	AMPLIFICATION	MODERATE	Gain	-

The *PALB2* frameshift mutations included a c.1592delT founder truncation mutation that has been previously identified as a breast cancer susceptibility gene in the Finnish population [19–20]. *PALB2* is a tumor suppressor gene which encodes a protein that stabilizes BRCA2 and allows to scaffold the molecular BRCA1-PALB2-BRCA2 complex at double-stranded breaks (DSBs) to prevent cells from accumulating DNA damage [21]. It thus plays a critical role in maintaining genome integrity through its role in the Fanconi anemia and homologous recombination DNA repair pathway, loss of which is a defining feature of the BRCAness phenotype and is associated with increased sensitivity to DNA damaging agents and poly-(ADP)-ribose polymerase inhibitors (PARPis) [22]. To assess sensitivity of the tumor cells to DNA damaging and other anti-cancer therapeutics, an *ex vivo* drug screening [15–17] of 165 drugs was initiated on the day of biopsy (Supplementary Data 1). Cells dissociated from the tumor tissue were exposed to the drugs for 96 hours and an enzymatic cell viability assay was used to assess cytotoxic drug effects with growth rate (GR) normalization [23] (Figure 1B). The measured cell-doubling rate of the tumor tissue derived cells for the GR method was ~760 hours corresponding to a low ~0.12 cell divisions over the course of the 96-hour screening assay. Comparison of the overall drug efficacy results (IC_50_) of the 165 drugs indicated that 48 drugs resulted in higher efficacy than any of the drugs that had been previously used to treat the patient (Figure 1B). 5 drugs with a known mechanism of action through targeting regulation of DNA damage responses (DDR) also displayed higher efficacy than the most potent previous used drug paclitaxel (Figure 1B). Of the DDR targeted drugs, two BETis JQ1 and ODM-207 were most potent with an IC_50_ of 1.33 and 2.12 μM respectively followed by DNAPKi NU7741 (IC_50_, 2.4 μM), WEE1i AZD1775 (IC_50_, 2.51 μM) and CHK1/2i AZD7762 (IC_50_, 4.95 μM) (Figure 1C). Estimated IC_50_ of olaparib was 36.33 μM displaying similar efficacy as the platinum-based drugs carboplatin and oxaliplatin (Supplementary Data 1).

### Patterns of BET and PARP inhibition efficacy on PALB2 altered cancer cell lines

**Figure 2:**
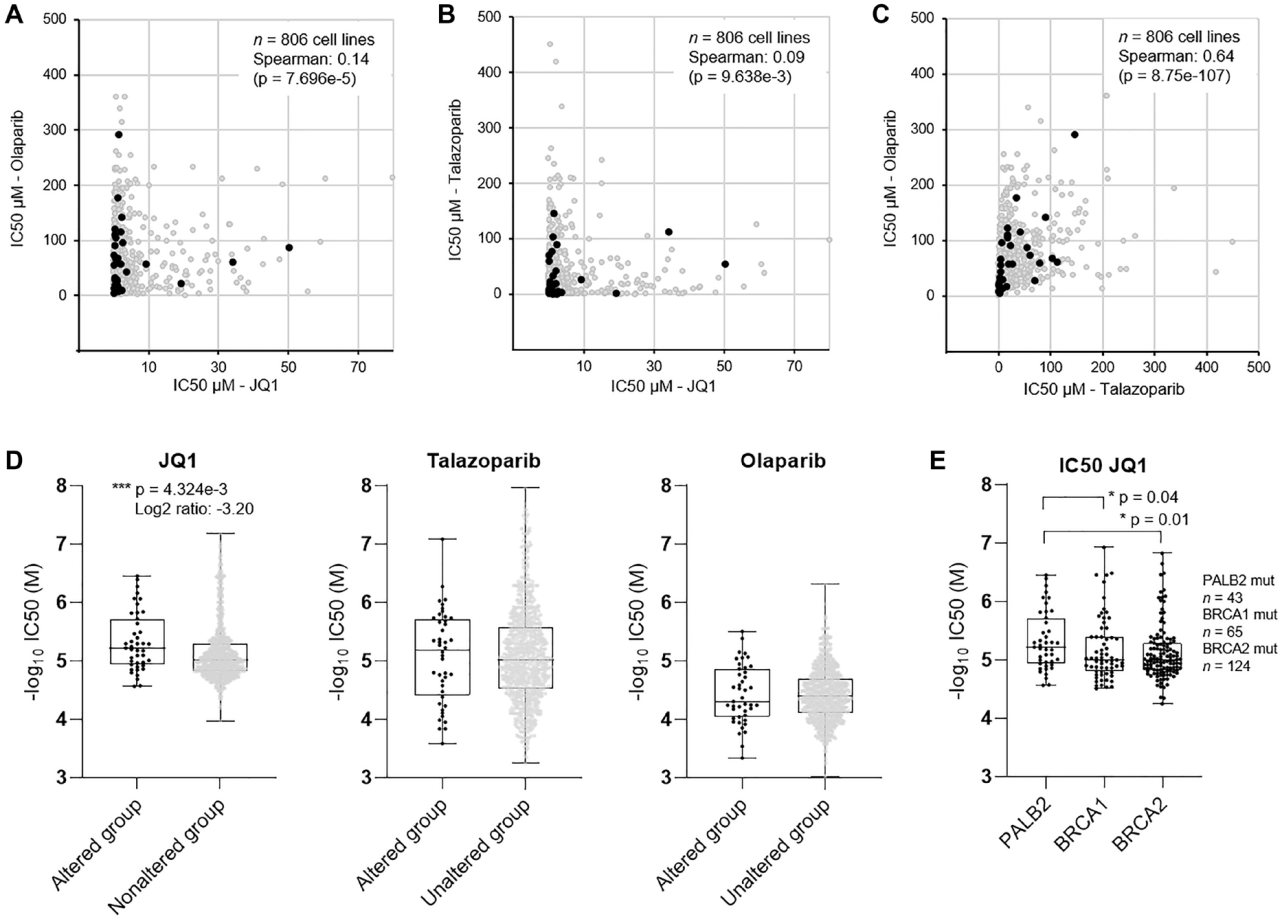
Evaluation of efficacy of PARPi and BETi in established human cancer cell lines. Scatter plots showing the pairwise correlation of IC_50_ as measure of potency of (**A**) olaparib and JQ1, (**B**) talazoparib and JQ1, and (**C**) olaparib and JQ1 in 806 cancer cell lines. Cell lines with alteration of *PALB2* shown in black. (**D**) Box plots showing the –log_10_ of IC_50_ (molar) of JQ1, talazoparib and olaparib in *PALB2* altered (*n* = 43) and non-altered (*n* = 763) cell lines. (**E**) Box plots showing the –log_10_ of IC_50_ (molar) of JQ1 in PALB2, BRCA1 and BRCA2 altered cell lines. *P* values derived from Student’s *t*-test.

The *ex vivo* drug screening results suggested substantial sensitivity of the patient cells to BETi and modest sensitivity to PARPi. The BRCAness phenotype and mutations in the core HR factors are both associated with increased sensitivity of cancer cells to PARPi [22] and BETi [24]. To evaluate if the efficacy of BETi and PARPi correlated in cancer cells with PALB2 alterations, we examined the efficacy of BETi JQ1 and two PARP inhibitors olaparib and talazoparib across 43 PALB2 altered 763 non-altered human cancer cell lines [25] with overlapping drug response data available from the Genomics of Drug Sensitivity in Cancer Project (GDSC2) (https://www.cancerrxgene.org) [26]. With comparison the drug efficacy on basis of IC_50_, the efficacy of both olaparib and talazoparib showed no correlation with JQ1 across all the cell lines (*p* = 0.00007 and *p* = 0.009 respectively) (Figure 2A and 2B), while efficacy of olaparib and talazoparib displayed a strong positive correlation (Spearman = 0.64, *p* = 8.75e-107) (Figure 2C). With comparison of the efficacy of PARPi and BETi on the PALB2 altered and non-altered cell lines, the efficacy of PARP inhibitors did not show statistically significant correlation with the PALB2 mutation status (Figure 2D). PALB2 altered cell lines however were found to be statistically significantly (*p* = 0.004) more sensitive to JQ1 than the non-altered cell lines (Figure 2D). The PALB2 altered cell lines were also found to be statistically significantly more sensitive to JQ1 than BRCA1 (*p* = 0.04) or BRCA2 (*p* = 0.01) altered cell lines (Figure 2E).

### *Ex vivo* DNA repair targeted RNAi screening

**Figure 3:**
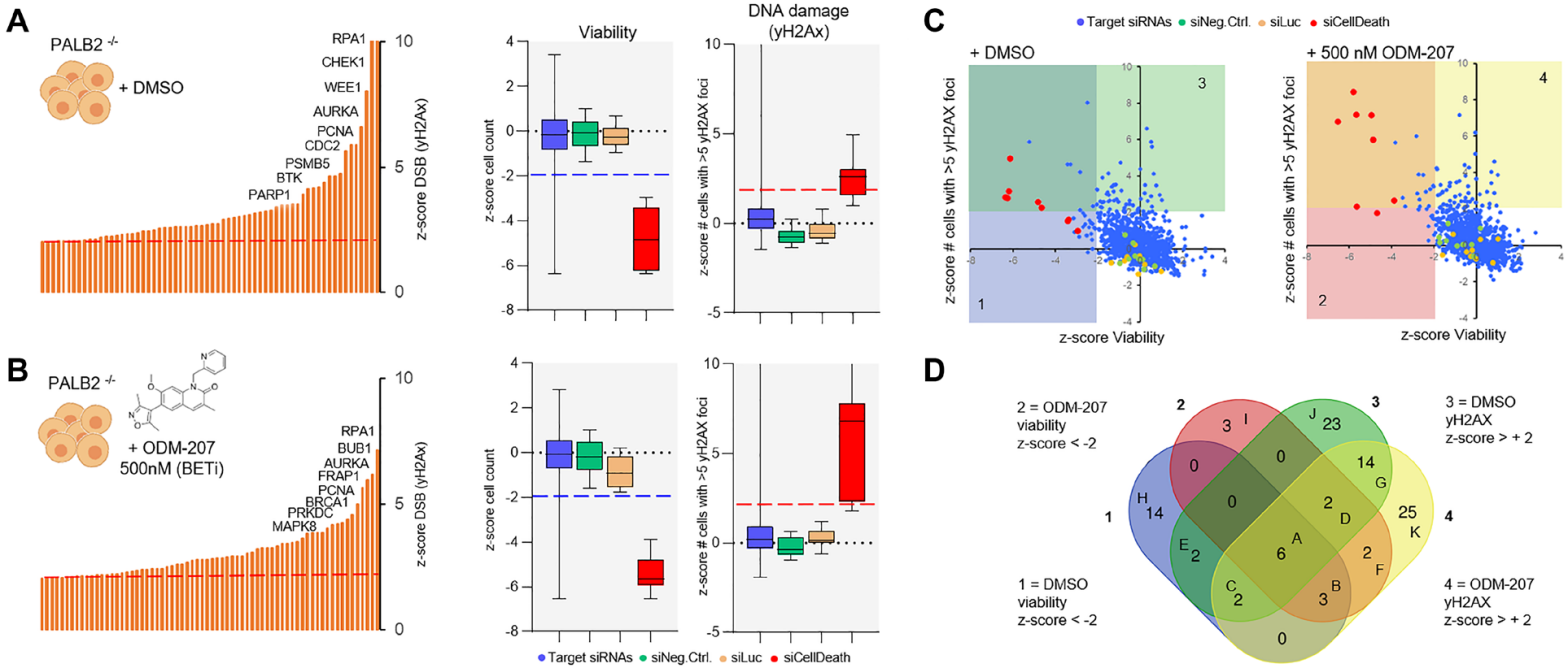
A DNA repair RNAi screen for the identification of essential DDR genes in the CAC cells. (**A**) In the control screen the cells were treated with DMSO and analyzed for viability on basis of cell counts and for induction of DNA damage on basis of quantification of nuclear γH2AX foci. Left; Bar graph distribution of siRNAs inducing an increase (z-score > +2) in the amount of nuclear γH2AX foci. 8 highest ranking genes shown in descending order. Right; Box plots showing the z-score distribution of all the target siRNAs and the control siRNAs for viability and DNA damage. (**B**) In the replicate screens cells were exposed to 500 nM ODM-207 before analysis of cell viability and DNA damage as above. (**C**) Scatter plots showing the correlation of the z-scores for viability and DNA damage in the control (left) and ODM-207 sensitization screen (right). The different siRNA classes shown with the indicated colors. (**D**) Venn diagram showing the overlap and distribution of the siRNA hits considered significant (z-score +/–2) for reducing cell viability or inducing DNA damage.

**Figure 4:**
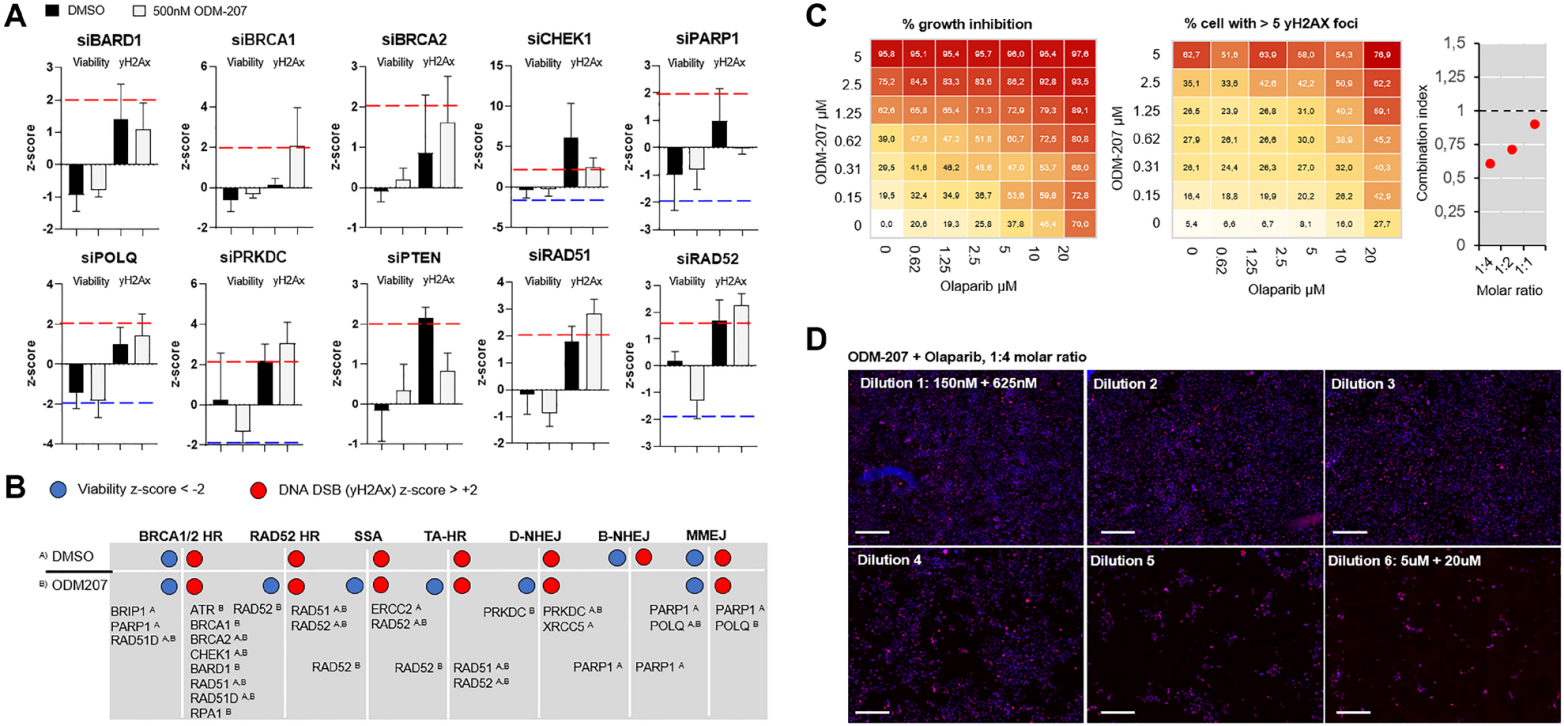
Analysis of efficacy of targeting different DNA repair pathways on *PALB2* mutated CAC cells. (**A**) Bar graphs showing the mean z-score and standard deviation of 3 individual siRNAs against core HR pathway genes. In each graph the left two bars show the viability z-score and right two bars the γH2Ax z-score in control condition (black) and with 500 nM ODM-207 (grey). (**B**) Distribution of the RNAi loss-of-function effects of the core HR genes divided according to the associated DNA repair pathway on untreated and BETi treated CAC cells. (**C**) ODM-207 and Olaparib exhibit a combinatorial additive effect on the PALB2 deficient cells. Dose–response matrix of percent of viability inhibition (left) and percent of cells with more than 5 nuclear γH2Ax foci (center) in response to increasing doses of ODM-207 (BETi) and Olaparib (PARPi). The combinatorial cytotoxicity was quantitatively analyzed by combination index (CI) combination index. With 1:4 molar ratio the CI50 of the drugs was 0.61, with 1:2 molar ratio 0.71, and with 1:1 molar ratio 0.89. (**D**) Representative 10× fluorescence microscopy images of the CAC cells stained for γH2AX (red) following 7d exposure to the combination of ODM-207 and Olaparib at 1:4 molar ratio. DNA staining shown in blue. Scale bars 100 μm.

To assess dependency of the patient derived cells on different DNA repair pathways and to identify DNA repair enzymes possibly contributing to the sensitivity or resistance of the cells to BETi, a siRNA screen with a custom library covering 300 genes with a known or suspected role in the DDR [18] was undertaken. Following successful validation of the ability to transfect the cells using a lipid transfection agent (Supplementary Figure 1), two replicate screens were initiated with the patient derived cancer cells remaining from the initial *ex vivo* drug screening. The first replicate screen was performed with DMSO control treatment (Figure 3A) while in the parallel replicate screen the cells were exposed to 500 nM ODM-207 for 48 hours (Figure 3B). Analysis of RNAi effects on cell viability were quantified on basis of cell counts, while the analysis of RNAi induced accumulation of DNA damage was performed with automated quantification of antibody detected nuclear γH2AX foci per cell (Figure 3A and 3B). In total 30 siRNA were found to significantly reduce viability of the cells and 64 induced an increase in DNA damage in the control screen (Figure 3C). 20 siRNAs reduced viability and 68 induced an increase in DNA damage in combination with the BETi ODM-207 (Figure 3C and 3D). Results of the siRNA screening are provided as Supplementary Data 2. RNAi inhibition of *AURKA*, *CHEK1* and *RPA1* were identified as the most potent siRNA hits (Supplementary Figure 1B) reducing viability and inducing DNA damage in both assay conditions supporting their key role in the regulation of DNA repair, replication and recombination including through promoting BRCA functions [22]. Most of the other siRNAs affecting the viability and genomic integrity of the cells were targeting genes with known roles in the different functions of HR and NHEJ pathways in context of replication fork stalling and restart (Supplementary Data 2). To evaluate dependency of the cells to specific DNA repair pathways rather than individual genes, we compared the effects of the core HR deficiency and BRCAness associated genes between the two replicate screens (Figure 4A). We found the cells to be collectively sensitive to targeting of the HR pathway, including core members *ATR, BRCA1, BRCA2, CHEK1, BARD1, RAD51* and *RAD51D* (Figure 4B). Inhibition of the core genes involved in the BRCA1/2-RAD51-dependent HR pathway sensitized the cells also to BET inhibition (Figure 4B). Targeting mediators of microhomology-mediated end joining (MMEJ) *POLQ* and *PARP1* (Figure 4A) also had a statistically significant viability reducing and DNA damage increasing effect on the cells supporting the concept of synthetic lethality of POLQ (polymerase theta, Polθ) inhibition in HR deficient cancers [27]. Targeting *RAD52*, a core mediator associated with transcription-associated homologous recombination repair (TA-HR), single strand annealing (SSA) and RAD52-RAD51-dependent HR on the other hand was synergistic with BETi (Figure 4A) supporting recent data suggesting synthetic lethality of dual targeting of RAD52 and the PARP1-mediated alternative NHEJ (B-NHEJ) [28]. Altogether, these results support the concept that BET-bromodomain inhibition promotes HR deficiency through depletion of the DNA double stand break resection protein CtIP (C-terminal binding protein (CtBP) interacting protein] [29]. The results also support that simultaneous inhibition of BET and PARP [22], as well as ATR, especially in context of *MYC* amplified cancers such as this case [30], might result in a robust synthetic lethality in HR-reduced cancer cells.

### BET and PARP inhibitor combination results in additive DNA damaging effect in PALB2 deficient cells

In the initial *ex vivo* drug screening the patient cells were identified to be sensitive to BETi. The *ex vivo* functional genetic screens indicated the cells to be sensitive to RNAi targeting of several component of the HR pathway, supporting that the therapeutic effects of BETi could be resulting from potentiation of the HR deficiency [24]. Targeting alternative DNA repair pathways including MMEJ and RAD52-mediated HR [28] on the other hand was synergistic with the BETi. The *in silico* evaluation of BETi and PARPi across hundreds of human model cell lines confirmed that the two drugs displayed independent efficacy, suggesting these drugs to have non-overlapping mechanisms of resistance and therefore low cross resistance [31]. We thus rationalized that combination of BETi and PARPi should potentiate the overall therapeutic efficacy of the two agents on the patient derived tumor cells. To test the hypothesis, we explored the effects of combining BET-bromodomain inhibitor ODM-207 and PARP inhibitor olaparib. The remaining patient derived cells were treated for 7 days with the two inhibitors, in a dose–escalating matrix to identify synergistic relationships of the two drugs (Figure 4C). At equal molar ratio of 1:1, the combination showed significant additive effect (CI_50_ Combination Index [32]: 0.89, IC_50_: 650 nM) on both cell viability and inducing DNA damage measured as number of nuclear DSBs (Figure 4C and 4D). The additive effect was significantly further potentiated with increasing the molar ratio of olaparib over ODM-207 to CI_50_ of 0.71 (IC_50_: 510 nM) at 1:2 molar ration and to CI_50_ of 0.61 at 1:4 molar ratio (IC_50_: 440 nM) (Figure 4D). These findings confirm that BETi, in combination with PARPi, results in synthetic growth inhibitory activity in PALB2 deficient cancer cells through induction of DNA damage accumulation resulting from the simultaneous blockade of compensating DNA repair processes [24].

### PALB2 mutations in non-melanoma skin cancers

BRCA mutations and the BRCAness phenotype are well known to be associated with breast, uterine, and ovarian cancers along with some non-gynecological malignancies involving the colon, prostate, pancreas and stomach. However, there are no reported cases to date of an association between cutaneous apocrine carcinomas or other non-melanoma skin cancers and familial BRCAness genetic background. Moreover, no systematic examinations of common shared genetic aberrations in the rare apocrine sweat gland cancers have been described. To assess frequency of mutations in the core HR associated genes or other DNA repair associated genes in non-melanoma skin cancers we examined all mutations of DNA repair gene ontology (term GO006281, *n* = 550 genes) associated genes across 687 non-melanoma skin cancer samples included in the AACR project GENIE database v 8.0 [33]. We found that 134 of the 550 DNA repair associated genes were mutated at least in one patient sample (Supplementary Figure 2A). *BRCA2* mutations were most frequent from the HR core genes with 12.1% (78/644) of analyzed samples harboring a mutation. *BRCA1* mutations were found 8.1% (52/644) of analyzed samples and *PALB2* mutations in 5% (32/635) of analyzed samples. Mutations of all; *PALB2, BRCA1, BRCA2* and *CHEK2* were most frequent in cutaneous squamous cell carcinomas covering ~60% of mutations in each of the genes (Supplementary Figure 2). Of the 687 non-melanoma skin cancer included in the dataset, four were sweat gland adenocarcinomas and ten samples were from a sweat gland carcinoma/apocrine eccrine carcinoma (Supplementary Figure 2C). No mutations in *BRCA1/2, CHEK2* or *PALB2* were detected in any of these samples leaving our patient as the first and only profiled cutaneous apocrine sweat gland carcinoma case with a *PALB2* mutation.

## DISCUSSION

Metastatic cutaneous apocrine gland carcinoma (CAC) is an extreme rare malignancy. Under 30 cases have ever been reported and the complex biology of these apocrine breast cancer-mimicking malignancies is poorly understood. Carriers of germline mutations in *BRCA1*, *BRCA2* and *PALB2* genes have been reported to have a higher risk of developing malignant abnormalities of the breast, colon, gynecological tissues, prostate and pancreas [22], but no association between germline mutations in these genes and non-melanoma skin cancers have been reported. In the current study, we identify a metastatic cutaneous apocrine sweat gland cancer with a biallelic *PALB2* mutation. The identified truncating *PALB2* c.1592delT mutation has been identified as a breast cancer susceptibility gene in the Finnish population with a prevalence of 0.2% and a 6-fold increased risk of breast cancer in the general population [19, 20, 34]. Also the c.470T>C (p.I157T) mutation in *CHEK2* in the patient’s tumor has been widely studied by us [35] and other in breast cancer predisposition in Finland and elsewhere. A study assessing *CHEK2* and *PALB2* mutations in high-risk Finnish BRCA1/2-founder mutation-negative breast and/or ovarian cancer individuals identified four cases of skin cancers with a co-occurring *PALB2* and *CHEK2* mutation [36], but unfortunately no details on the type of these cancers is included. In functional molecular biology studies, the *PALB2* c.1592delT mutation has been shown to channel DNA double-strand break repair into error-prone pathways in breast cancer patients suggesting increased dependency of these cancer to NHEJ, MMEJ and SSA (single strand annealing) and SSA-HR to compensate for the HR deficiency [37]. The *ex vivo* drug and RNAi screens performed on the patient derived cells confirmed synthetic lethality of targeting these pathways with both siRNA and drug mediated inhibition. The Finnish Medicinal Agency (FIMEA) granted an approval for use of talazoparib on compassionate-use basis for the patient. Unfortunately, the talazoparib treatment was stopped after first round of therapy due to rapid worsening of the patient’s general health condition and no objective evaluation of the response to therapy could be established. The patient was started on best palliative care and he succumbed to the disease shortly after stopping of the active treatments.

Although the efforts here ultimately did not result in successful treatment of the patient, the significance of our study is its demonstration that this type of functional *ex vivo* analyses if performed in the early stages of disease could provide valuable insights into treatment of rare cancers where there is limited data available to base treatment decisions on. Our analyses of sensitivity of PALB2 deficient cancer cells to inhibition of the different DNA repair pathways also offer a valuable data resource for testing and building new hypothesis on. In summary, by interrogating the patient-derived cells, we identify a potential therapeutic opportunity for targeting PALB2 deficient cells through inhibition of DNA repair with BETi or PARPi. Moreover, we identify the *PALB2* c.1592delT mutation as a potential susceptibility factor for non-melanoma skin cancer in the high cancer risk families carrying this founder mutation.

## MATERIALS AND METHODS

### Patient and tissue specimens

The patient, a 45-year old male, was identified to the study following progression of the disease after the standard therapeutic options had been exhausted. In context of the primary diagnosis of the patient’s tumor as a metastatic cutaneous apocrine sweat gland carcinoma (CAC), the immunohistochemistry profile of the tumor tissue had been AR^+^. ER^+^, PR^-^, HER2^-^, CK7^+^ [4]. GCDFP15^-^ and mammaglobin^-^ (SCGB2A2) [5] with Ki67 index of 40%. Metastases were inoperable and patient was referred for systemic therapy. First line chemotherapy consisted of docetaxel followed by docetaxel-capecitabine combination therapy resulting in a sustained clinical response. Following recurrence, the treatment was changed to CEF regimen (cyclophosphamide/epirubicin/fluorouracil) resulting in disease stabilization. After second recurrence, treatment was changed to docetaxel-gemcitabine, followed by capecitabine-vinorelbine regimen, followed by paclitaxel-carboplatin regimen and finally with eribulin. Whole-body CT (computerized tomography) scan indicated no response to any of these treatments and hormonal therapy was attempted with tamoxifen and bicalutamide. The disease showed clear metastatic progression with no therapeutic benefit from the hormonal therapy. The patient was then considered for detailed molecular pathology profiling and the *ex vivo* therapy sensitivity study. As per discussion with the patient and with approval from the local Ethics Committee of the Helsinki University Hospital, needle biopsy samples were collected for the *ex vivo* drug screening and DNA sequencing from a subcutaneous metastatic lesion in the neck. Altogether five 18-gauge coarse needle biopsy cores were collected. All the experiments were undertaken with the understanding and written informed consent of the patient and the study methodologies conformed to the standards set by the Declaration of Helsinki.

### Tumor derived primary cell culture

Four coarse needle biopsy cores sampled from the metastatic lesion were devoted to establishing a vital cell culture from the patient’s tumor cells and one core was sent for DNA extraction and next generation sequencing. The remaining tissue cores were placed in sterile RMPI-1640 medium (Gibco) without supplements for transport to the research laboratory (Figure 1A). Immediately upon receipt, the live tissue samples were processes into a single cell suspension as previously described [15]. The suspension was diluted to RPMI-1640 medium supplemented with 1% FBS, 1x Insulin-Transferrin-Selenium supplement (ITS-G, Gibco) and penicillin-streptomycin to achieve a suspension with ~500 cells per 45 μL of medium. In total ~3 × 10^5^ cells isolated from two 18-gauge needle cores were used for the drug screen and the rest were placed to cell culture in the above medium in standard cell culture conditions (37°C, 5% CO_2_).

### Next generation sequencing

Targeted DNA sequencing using FoundationOne^®^ CDx test was purchased as a service from Foundation Medicine, Inc., with diagnosis submitted by the clinician. Results of the analysis are provided in the Table 1.

### *Ex vivo* drug screening

The primary drug screening was performed as previously described [15–17]. Briefly, the therapeutic compound collection consisted of 165 drugs (Supplementary Data 1) in four concentrations adjusted separately for the different drugs and readily printed onto tissue culture treated 384-well microplates (Corning). A single-cell suspension of freshly isolated tumor cells (45 μl per well; 300 cells per well) was transferred to each well using a peristaltic Multidrop reagent dispenser (ThermoScientific). The 384-well plates were incubated for 96 h at standard cell culture conditions (37°C, 5% CO_2_). Analysis of cell viability was performed with assessment of total adenosine tri-phosphate (ATP) levels in living cells using CellTiter-Glo luminescent cell viability assay reagent (Promega). Luminescence was measured using a Labrox multilabel microplate reader (Labrox). The luminescence signals were normalized to DMSO-only wells (negative control), 5 μM staurosporin-containing wells (positive control) and 2 μM aphidicolin-containing wells (cell growth control) to allow for growth rate normalization of the dose responses [23]. The validation drug screen experiments were performed using imaging cytometry [15]. Briefly, cell cultures were treated with increasing concentrations of olaparib and ODM-207 as a combination for 7 days. Cell were fixed using 4% paraformaldehyde with 0.3% Triton-X100 and stained with an antibody to detect phosphorylation of γH2Ax (pS139) (Abcam, ab2893) to allow measurement of nuclear DNA double strand breaks (DSBs). Primary antibody was labelling was performed with a goat-anti-rabbit Alexa647 conjugated secondary antibody (Molecular Probes) and DAPI (1 μg/ml) was used for DNA counterstaining. Cells were imaged with an Olympus scan^R platform at 20× magnification. 6 frames were acquired from each 384-well and the images were analyzed with Olympus scan^R image analysis suite (Olympus-SIS). The Supplementary Data 1. including the drug screen data has been deposited to Mendeley Data (https://doi.org/10.17632/yy68tb6fd4.1).

### *Ex vivo* RNAi screening

The phenotypic image-based siRNA screens were performed in 384-well format as previously described [18]. Briefly, a custom collected DNA damage siRNA library consisting of three individual siRNAs for 300 DNA repair genes (ON-TARGET plus siRNA, Dharmacon) was pre-printed into 384 wells and complexed with 0.06 μL of siLentFect (Bio-Rad) lipid transfection agent for transfection (Supplementary Figure 1). 2000 patient derived tumor cells in 40 μL of media was applied onto each 384 well resulting in 25 nM final siRNA concentrations and allowed to transfect for 24 hr. Replicate one screen plates were treated with 0.05% DMSO and replicate two plates with 500 nM ODM-207 (Orion Pharma, Turku, Finland). Due to the limited number of cells obtained from the tissue biopsies, no biological replicate experiments were possible. After 48 h exposure the cells were fixed and stained as detailed above with an antibody for γH2AX (pS139) and DAPI for DNA. Immunostained cells were imaged and analyzed using the Olympus scan^R high content imager. Integrated nuclear DNA staining was used for imaging cytometry and nuclear γH2AX foci counts per nuclei were quantified using a watershed object identification algorithm. For analysis of significance, a z-score was calculated for total cell counts as measure of viability and for cells with more than five nuclear γH2AX foci as measure for induction of DNA damage. From this analysis, siRNAs with z-scores ± 2 standard deviations (compared to plate mean and standard deviation of all siRNAs including controls) were considered significant. The Supplementary Data 2. Including the RNAi data has been deposited to Mendeley Data (https://doi.org/10.17632/yy68tb6fd4.1).

### *In silico* cancer genomics analysis

Assessment of frequency and cancer type level distribution of genomic aberrations the DNA repair associated genes (gene ontology term GO006281, DNA repair) was performed with data from the AACR Project GENIE dataset v 8.0 (Genomics Evidence Neoplasia Information Exchange) via the cBioPortal (http://www.cbioportal.org) on July 15, 2020.

### Statistical analysis

The *ex vivo* drug screening data was analyzed using the normalized growth rate inhibition (GR) approach which yields per-division metrics for drug efficacy. Dose response curves for growth rate normalized IC_50_ estimates were generated in GraphPad Prism software (V8, GraphPad Software Inc.). Combination indices (CI) were calculated from the fixed-ratio, dose escalation experiments using the Chou and Talalay method [32]. CI values were reported at 50% inhibitory values (CI_50_). Welch’s *t*-test, Spearman and Pearson correlation analyses were applied using GraphPad Prism V8 software as indicated in the figure legends according to assumptions on data normality.

## SUPPLEMENTARY MATERIALS



## Abbreviations

BET: Bromodomain and Extra-Terminal
CAC: Cutaneous apocrine carcinoma
DDR: DNA Damage Response
DSB: Double strand break
HR: homologous recombination
MMEJ: Microhomology-mediated end joining
NGS: Next generation sequencing
NHEJ: Nonhomologous end joining
PALB2: Partner and localizer of BRCA2
PARP: poly ADP ribose polymerase
RNAi: RNA interference
