# Supplementary material for: Ex vivo analysis of DNA repair targeting in extreme rare cutaneous apocrine sweat gland carcinoma
Source: Oncotarget. 2021 May 25;12(11):1100–9. doi: 10.18632/oncotarget.27961 (PMC8169071; doi:10.18632/oncotarget.27961)
Supplement: Supplementary file 1 [file oncotarget-12-1100-s001.pdf]

Ex vivo analysis of DNA repair targeting in extreme rare cutaneous apocrine sweat gland carcinoma

SUPPLEMENTARY MATERIALS

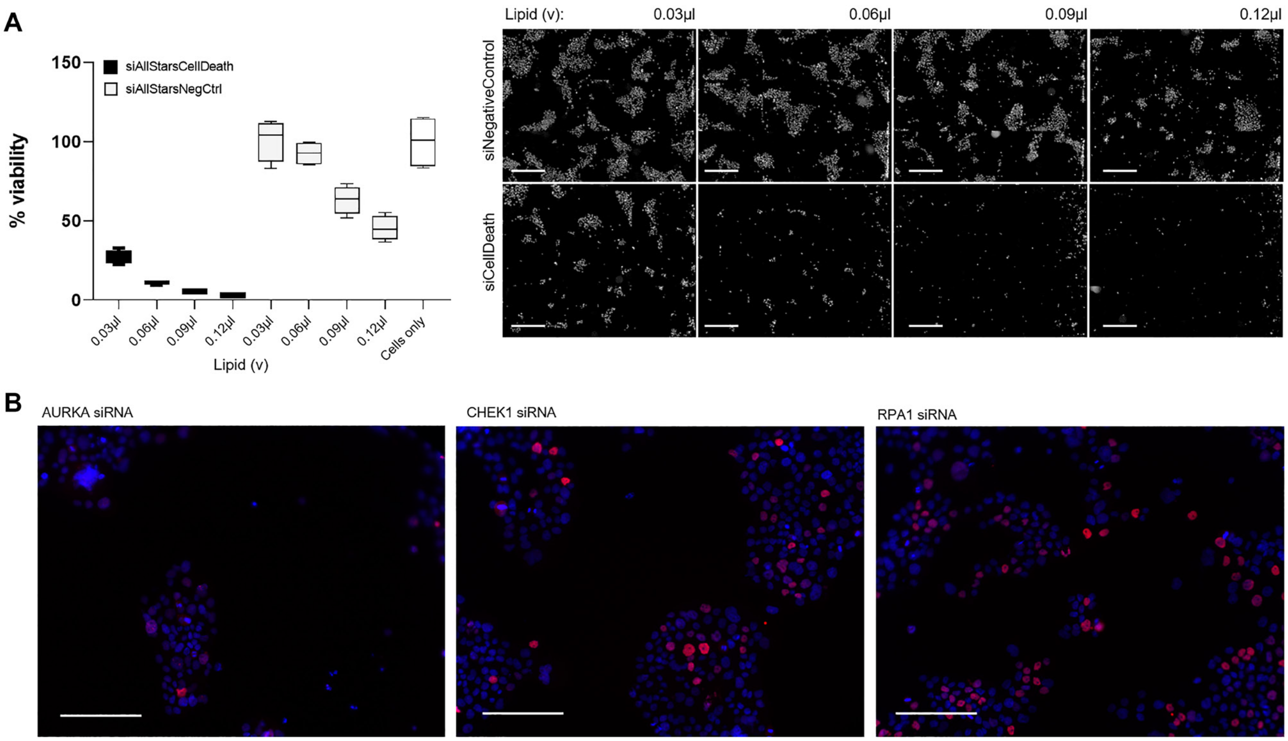

Supplementary Figure 1: (**A**) Optimization of transfection conditions with different volumes of the lipid transfection agent and 25 nM siRNAs. Transfection efficacy was determined on basis of cell killing efficacy of the CellDeath control siRNA in comparison the negative control scrambled control siRNA. Right; Representative 10× fluorescence microscopy images of the cells after 72 h transfection stained with DAPI DNA stain. Scale bars 100 µm. (**B**) Representative 20× fluorescence microscopy images of the CAC cells transfected with the indicated siRNAs. γH2Ax staining shown in red, DNA in blue. Scale bars 50 µm.

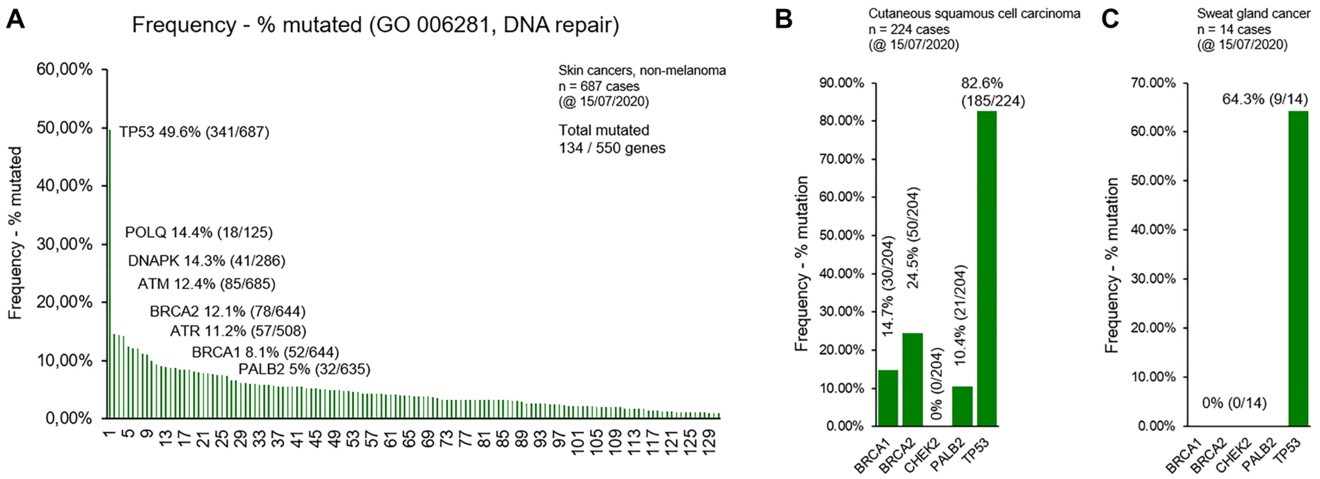

Supplementary Figure 2: Frequency of BRCAness associated genomic alterations in non-melanoma skin cancers. (**A**) Histogram plot generated from AACR Project GENIE 8.0 data (Genomics Evidence Neoplasia Information Exchange) available at cBioPortal (http://www.cbioportal.org) showing the frequency of mutations of any DNA repair genes (GO term 006281) across different types of non-melanoma skin cancers ($n = 687$). Mutations of 134 DNA repair associated genes were present in at least 1 patient sample. (**B**) In non-melanoma skin cancers mutations of *BRCA1, BRCA2, CHEK2* and *PALB2* are most frequent in cutaneous squamous cell carcinomas with mutations in *BRCA2* identified in 50 of 204 profiled tumor samples. (**C**) No mutations of *BRCA1*, *BRCA2*, *CHEK2* or *PALB2* have been identified from the 14 analyzed sweat gland cancers.

Supplementary Data 1: *Ex vivo* drug screening results. https://doi.org/10.17632/yy68tb6fd4.1.

Supplementary Data 2: *Ex vivo* RNAi screening results. https://doi.org/10.17632/yy68tb6fd4.1.
